# Self-Reset Image Sensor With a Signal-to-Noise Ratio Over 70 dB and Its Application to Brain Surface Imaging

**DOI:** 10.3389/fnins.2021.667932

**Published:** 2021-06-15

**Authors:** Thanet Pakpuwadon, Kiyotaka Sasagawa, Mark Christian Guinto, Yasumi Ohta, Makito Haruta, Hironari Takehara, Hiroyuki Tashiro, Jun Ohta

**Affiliations:** ^1^Division of Materials Science, Graduate School of Science and Technology, Nara Institute of Science and Technology, Takayama, Japan; ^2^Division of Medical Technology, Department of Health Sciences, Faculty of Medical Sciences, Kyushu University, Maidashi, Japan

**Keywords:** self-resetting, CMOS image sensor, implantable device, high signal-to-noise ratio, *in vivo* experiment, intrinsic signal, somatosensory cortex, image processing

## Abstract

In this study, we propose a complementary-metal-oxide-semiconductor (CMOS) image sensor with a self-resetting system demonstrating a high signal-to-noise ratio (SNR) to detect small intrinsic signals such as a hemodynamic reaction or neural activity in a mouse brain. The photodiode structure was modified from N-well/P-sub to P+/N-well/P-sub to increase the photodiode capacitance to reduce the number of self-resets required to decrease the unstable stage. Moreover, our new relay board was used for the first time. As a result, an effective SNR of over 70 dB was achieved within the same pixel size and fill factor. The unstable state was drastically reduced. Thus, we will be able to detect neural activity. With its compact size, this device has significant potential to become an intrinsic signal detector in freely moving animals. We also demonstrated *in vivo* imaging with image processing by removing additional noise from the self-reset operation.

## Introduction

The nervous system controls significant body function. It is particularly important to understand its dynamics and functionality to prevent or cure diseases that affect the brain (Hillman, 2007; Haruta et al., 2019). Although numerous methods have been developed to offer various strategies to study brain function, they remain poorly understood because of their complexity, and each method has its limitations. Optical imaging is an important approach for studying the brain. Cell level imaging of an exposed cortex can be performed using a scanning microscope, such as confocal or two-photon microscopy (Yoder and Kleinfeld, 2002; Hillman, 2007; Wilson et al., 2007; Takehara et al., 2014). However, it is not suitable for a wide field of view. Other than that, fluorescence microscopy is a popular brain imaging technique (Grinvald and Hildesheim, 2004; Jung et al., 2004; Wang et al., 2008). All these methods face the same problem. The experimental setup with the microscope made it impossible to study the animal while it is freely moving or demonstrating natural behavior. Observing the brain of an animal behaving naturally is essential in the study of the mechanisms that underlie its function. To overcome these limitations, several groups have developed miniaturized devices (Ferezou et al., 2006; Sawinski et al., 2009; Ghosh et al., 2011; Helmchen et al., 2001). These are still based on conventional microscope optics. The required equipment obstructs the animals’ natural behavior. An implantable optical imaging device based on complementary-metal-oxide-semiconductor (CMOS) technology is an alternative approach to observe neural activities while moving freely (Ohta et al., 2009, 2017; Haruta et al., 2015; Takehara et al., 2015). Previously, our group has reported implantable CMOS image sensors for contact imaging, which are ultra-small and lightweight. Thus, it is suitable for implantation (Ng et al., 2006; Tamura et al., 2008; Haruta et al., 2013, 2014, 2015).

To realize an alternative method for study of a mouse brain without relying on an additional step or preparing the sample with genetic engineering, intrinsic signal imaging was chosen. These signal intensity changes are correlated with different brain states. However, some important reactions in the mouse brain only produce an exceedingly small signal change. Thus, a high performance is required to detect changes of approximately 0.1% in intrinsic brain signals. A high signal-to-noise ratio (SNR) greater than 60 dB is required to monitor these small signals. The peak SNR of a normal active pixel sensor (APS) is typically 40–50 dB (Ohta, 2017). In our previous work, we proposed the use of a self-resetting pixel to achieve a high effective SNR and succeeded in obtaining a high SNR of 64 dB, which can detect the hemodynamic signal from the mouse brain along with the stimulation (Sasagawa et al., 2016a, b; Yamaguchi et al., 2016). However, the experiment could still be improved. It has a chance to receive a better high-definition signal from the brain if the device has a sufficiently high effective SNR. We found that the active circuit used to connect to the V_*rst*_ has insufficient stability and may cause a signal drop or significantly increase the noise by approximately 3 dB at the first reset. Subsequently, it becomes a floor for the overall SNR. To improve the effective SNR, this voltage drop must be minimized.

**FIGURE 1:**
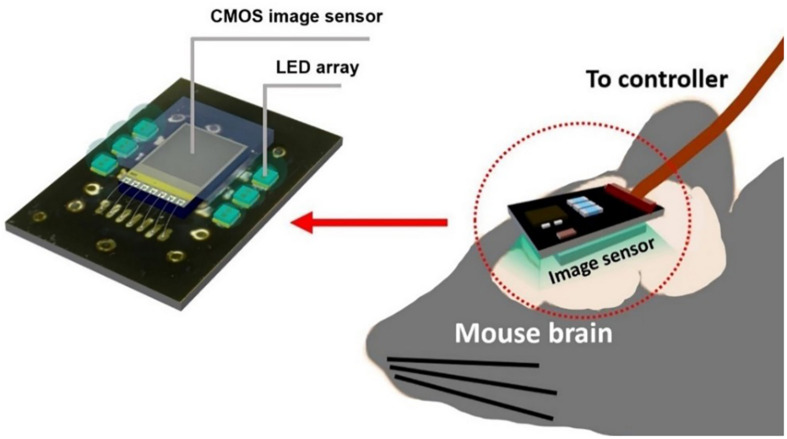
Diagram of an implantable self-reset image sensor for detecting an intrinsic signal from hemodynamics reaction. The proposed image sensor was attached with the relay board surrounded by LEDs.

To gain an even higher effective SNR, we introduce a modified photodiode structure to increase the photodiode capacity. P-diff/N-well/P-sub was chosen as a photodiode because it has a higher capacity owing to its physical structure having more thin layer area than an N-well/P-sub of the same size. This allows the pixel to handle more electrons within one resetting cycle. In short, it reduces the number of self-resets and avoids the unstable stage. We also used a new relay board with improved performance. Moreover, we developed image processing to reduce artifacts from the self-resetting system. The device implementation concept is illustrated in Figure 1.

This article is organized as follows. In section “Self-Reset Imaging Device,” we present the proposed pixel circuit, which explains the basic principle, self-reset pixel concept, and the photodiode structure used in this image sensor. Subsequently, the device and its smart relay board fabrication and process are explained. In section “Imaging Device Characteristics,” the characteristics of the imaging device are shown. In section “Imaging Experiment,” the *in vivo* experimental setup and the image processing procedure are described. Section “Discussion” is for the discussion, touching on the comparison with other sensors, limitations of the device, and the imaging results. After the conclusion in section “Conclusion”, the following sections are the additional information, which contains the declaration of conflict of interests, author contributions, and funding.

## Self-Reset Imaging Device

### Image Sensor

#### Operational Principle

The self-reset image sensor detects the charge accumulated in the pixel and resets the pixel by itself before saturation occurs. As a result, the effective pixel capacity can be increased, and the amount of manageable light can be increased. One of its applications is high-dynamic-range imaging. However, our purpose was to obtain a highly effective SNR. Under high light intensity, photon-shot noise is the dominant noise factor. Its value is proportional to the square root of the amount of incident light, and to achieve a high SNR, it is necessary to avoid pixel saturation and manage a large number of photo carriers. Before discussing the high SNR in the self-resetting sensor, we need to define the parameter that limits the effective SNR. Under high-intensity conditions, photon shot noise is the primary noise. The total self-reset sensor noise σ_*SELFRST*_ is approximately,

(1) $$\sigma_{SELFRST}≃\sigma_{SN}\sqrt{\frac{\sigma_{RST}^{2}}{FWC}+1},$$

where σ_*RST*_ is the pixel reset noise, σ_*SN*_ is the photon shot noise, and *FWC* is the full-well pixel capacity. In the case of a sufficiently high *FWC*, the total noise asymptotically approaches σ_*SN*_. The signal components are shown in Eq. 2,

(2) $$V_{sig}=V_{out}+N\cdot A_{out}.$$

From Eq. 2, *V*_*out*_ is the pixel output, *N* is the number of resetting cycles, and *A*_*out*_ is the maximum amplitude of the pixel. The effective SNR is described by Eq. 3.

(3) $$SNR_{eff}=\frac{V_{sig}}{\sigma_{SELFRST}},$$

According to Eqs 1 and 3, we found that the self-reset sensor SNR is nearly the same as that of a normal image sensor under a high light intensity condition. Because the self-resetting system prevents the pixel from saturation, *V*_*sig*_ can be as high as the proportional light intensity. After the post-processing for signal reconstruction, the intensity signal can be retrieved by compensating it with an estimated number of resetting cycles. In this demonstration, the number of self-resetting *N* is unnecessary, especially when the intensity changes are significantly small. One advantage of self-resetting pixels is their effective high dynamic range. However, in this study, we focused on detecting small signals, such as the change from the intrinsic brain signal, where a high dynamic range helps to reach a sufficient SNR.

#### Pixel Circuit

**FIGURE 2:**
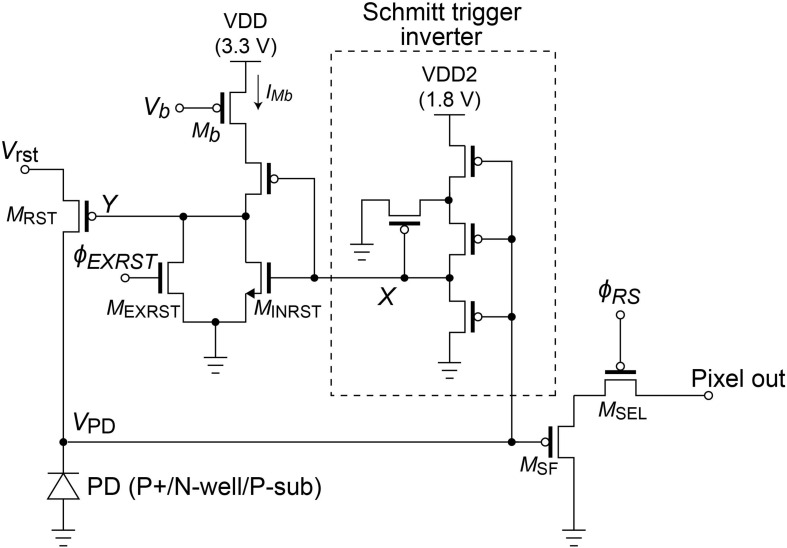
Schematic of the self-reset pixel with a low-voltage driven Schmitt trigger inverter. The PD is composed of P+/N-well/P-sub.

The entire circuit design is still based on a previous version (Sasagawa et al., 2016a, b; Yamaguchi et al., 2016). We still use the same 4-transistor Schmitt trigger inverter as in the previous version. It is driven by a lower voltage VDD2 than the other portions to reduce power consumption. The pixel circuit integrated with the Schmitt trigger inverter is shown in Figure 2.

**FIGURE 3:**
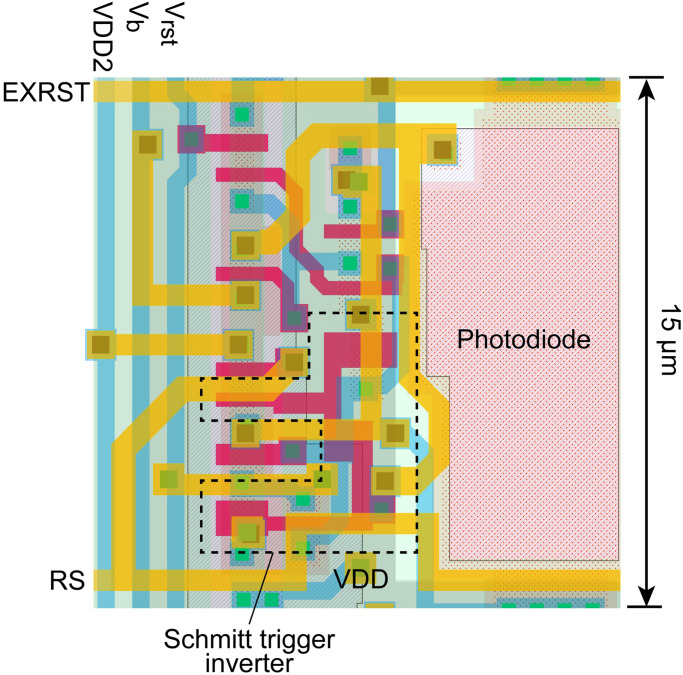
Layout of the pixel with P+/N-well/P-sub photodiode.

The layout of the proposed pixel with a photodiode is shown in Figure 3. In this study, the photodiode type was composed of P+/N-well/P-sub. This structure increases the photodiode capacitance compared to the standard N-well/P-sub structure, which is usually used in 3-transistor APS pixels. However, the pixel size can still be maintained at 15 μm × 15 μm. Eleven transistors were used in the pixel, which is the minimum number for this process.

#### Pixel Simulation

**FIGURE 4:**
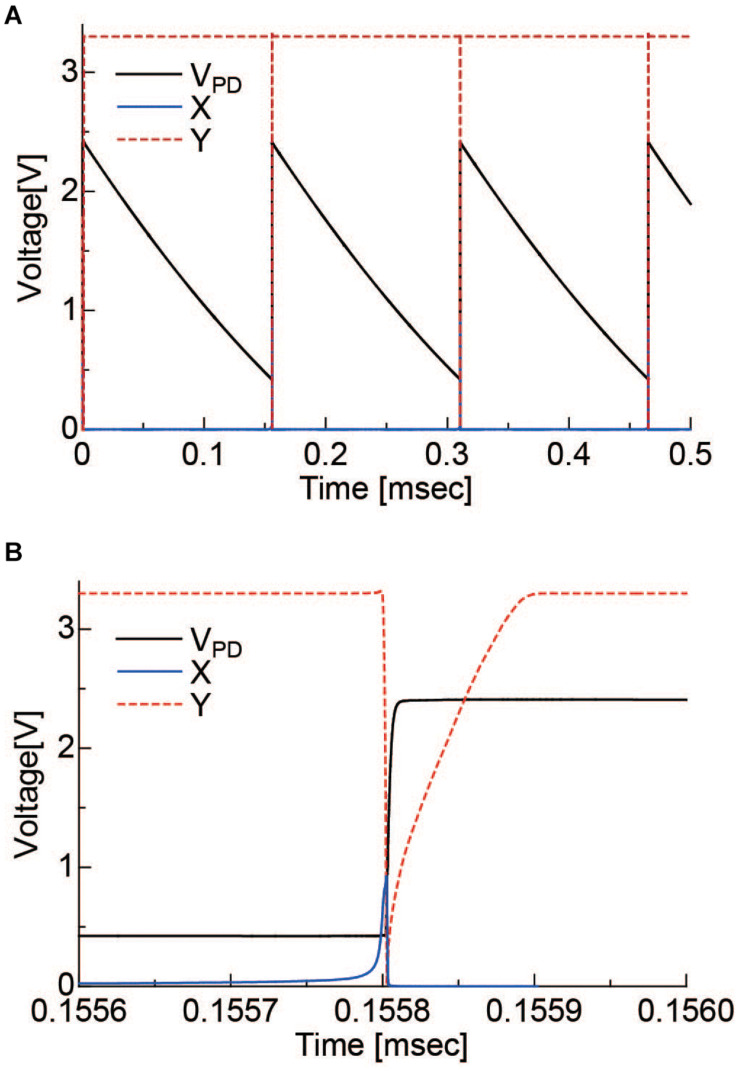
**(A)** Simulation results of the self-resetting pixel. **(B)** is the magnified plot of the self-resetting region. X, Y correspond to the nodes indicated in Figure 2.

The operation of the pixel circuit was simulated. V_*rst*_, V_*B*_, and VDD2 were set to 2.4, 2.5, and 1.8 V, respectively. In addition, a constant current source of 1 nA was placed in parallel with the PD to simulate the PD photocurrent. The results are shown in Figure 4. Figure 4A is the period during which several self-resets occur, and Figure 4B is the magnified self-reset period plot. After the PD is charged to V_*rst*_ by an external reset, the V_*PD*_ is gradually lowered by the photocurrent. When it reaches the Schmitt trigger inverter threshold, node X in Figure 2 is inverted and becomes HIGH. Furthermore, it is inverted by the inverter circuit, and the reset signal of node Y becomes LOW. The period of this LOW state was approximately 100 ns. The optical signal could not be detected during this period. When the frame rate is 30 fps and the number of self-resets is 20 times per frame, the output change during this period is 1/1.67 × 10^4^ of the V_*PD*_ voltage swing. Under this condition, the SNR exceeds 70 dB, as discussed in section “Pixel Output.” Because the photon shot noise, which is the dominant temporary noise, is estimated to be approximately 1/3 × 10^3^, which indicates that the self-reset time is sufficiently short in this case.

#### Image Sensor Chip

**FIGURE 5:**
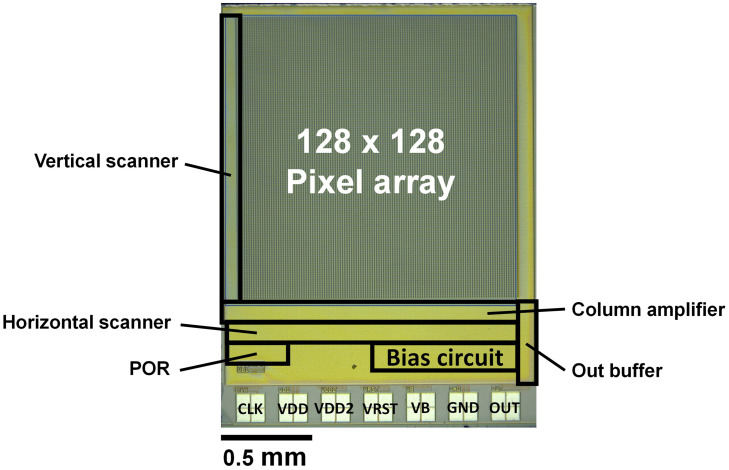
Graphic of the proposed image sensor.

A graphic of the fabricated chip is shown in Figure 5. We used the TSMC 0.35-μm 2-poly 4-metal standard CMOS process. The specifications are listed in Table 1. The pixel array is 128 × 128 pixels. The basic configuration of this image sensor is the same as that of our implantable image sensor, and the control line is reduced by generating a control signal from an external clock. However, the analog signal line is externally input. As a result, there are seven signal lines. In particular, the reset voltage (VRST) decreases during self-reset, but this must be minimized. It is difficult to mount a sufficient performance bias inside the chip. The image sensor output is an analog voltage signal.

**TABLE 1:** Chip specifications.

Technology	TSMC 0.35 μm 2-poly 4-metal standard CMOS process
Chip size	2.7 × 2.1 mm^2^
Pixel size	15 × 15 μm^2^
Photodiodes	P+/N-well/P-sub
Full well capacity	0.72 Me^–^
Fill factor	30%
Pixel number	128 × 128
Operating voltage	3.3 V
Pixel type	3-Tr active pixel sensor with 4-Tr Schmitt trigger inverter for self-resetting

### Fabricated Device

#### Relay-Board

**FIGURE 6:**
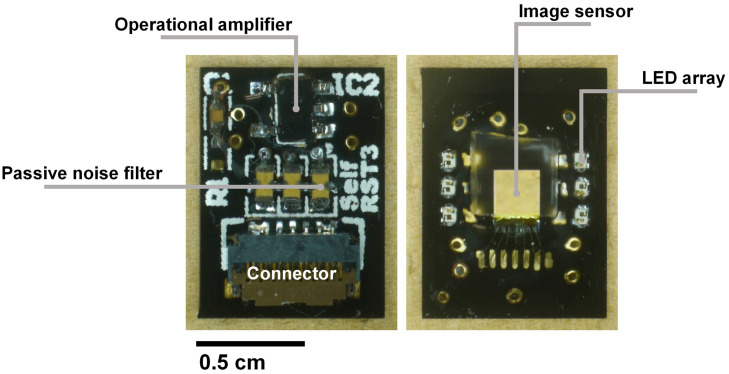
Images of the relay board **(left)** with the preamplifier circuit and passive noise filters placed on this side. **(Right)** The image sensor is mounted between the LED arrays to benefit from the high light intensity. The image sensor surface was covered by the fiber optic plate and sealed with epoxy resin for waterproofing.

This custom printed circuit board (PCB) connects the image sensor to the data processing board. Because the image sensor output is an analog signal, it is equipped with a preamplifier circuit. As mentioned in the previous section, the V_*RST*_ must minimize the voltage drop amplitude and duration as a result of self-reset. Because it is difficult to obtain a sufficient response speed in an active circuit, passive noise filters were inserted into the V_*RST*_ line on this board. On the other side, the image sensor and six LEDs were mounted as shown in Figure 6.

#### Device Assembly

Because the target application is to be implanted in a mouse brain, we must consider both the thermal condition and waterproof packaging. After connecting the Al wires, epoxy was used to protect all electrode surfaces. Incidental light from the side of the image sensor causes artifacts. To prevent this, a black-colored resist was applied between the LEDs and the sensor. The image sensor was covered with a fiber optic plate (FOP, J5734, Hamamatsu). The FOP is an optical device consisting of a bundle of microoptical fibers. It directly conveys an incident image on its input surface to its output surface. The thickness is 500 μm. This FOP protects the image sensor surface and maintains the distance between the brain surface and the mounted LED because damage owing to its heat may be caused.

## Imaging Device Characteristics

### Pixel Output

**FIGURE 7:**
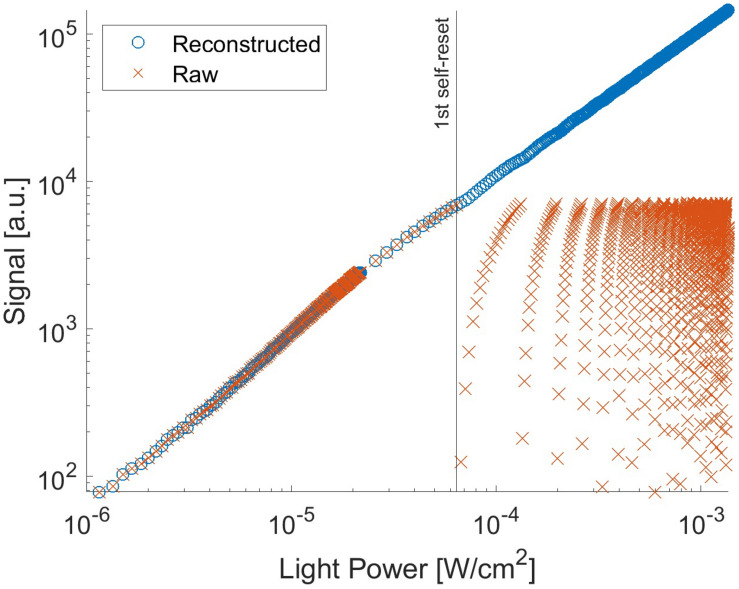
Signal from the pixel with P+/N-well/P-sub photodiode as a function of the light intensity.

With the proposed pixel, we set it up as follows: the V_*b*_ can control the current flowing through M_*b*_. Thus, the self-resetting time can be adjusted. We optimized it at 2.5 V with a reset duration of approximately 0.1 μs. Figure 7 shows the output signal as a function of light intensity. In this measurement, the chip was illuminated by a uniform beam with a peak emission wavelength of 530 nm. The signal was observed for one selected pixel. The evaluation was performed at room temperature. When the pixel voltage V_*PD*_ shown in Figure 2 becomes lower than the Schmitt trigger inverter threshold, a self-reset is triggered. Thus, the output signal is reset to zero. As a result, the output shape is similar to that of a saw tooth. However, the real illuminated signal can be easily reconstructed when the number of self-resets is known. The reconstructed signal output is plotted in Figure 7. For reconstruction, the output amplitude and estimated number of self-resets were added to the signal. From this result, the prototype device has nonlinearity with respect to the light intensity, and the difference in slope is large, especially just before and after the self-reset. Therefore, in the imaging experiment using this device, a look-up table was prepared based on this result, and correction was performed by image processing.

**FIGURE 8:**
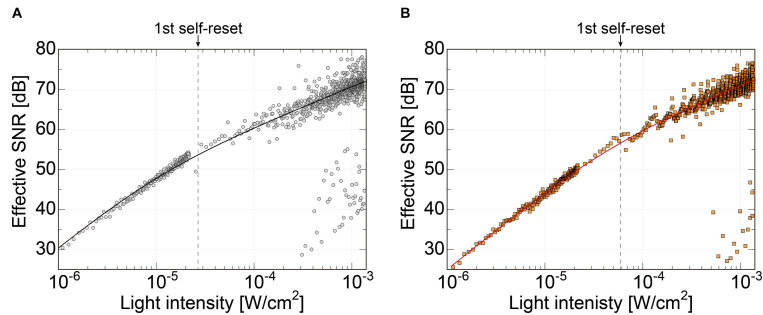
**(A)** SNR of the pixel with N-well/P-sub photodiode, **(B)** SNR of the pixel with P+/N-well/P-sub photodiode.

Figure 8 shows the effective SNR as a function of illuminated light power. It was calculated using Eq. 3. Figure 8A shows the result of the previous N-well/P-sub structure, and Figure 8B shows that of the P+/N-well/P-sub structure. The plot points with a low SNR appear in the high light intensity region, which is at the boundary of the number of self-resets. This can be reduced by correcting the artifacts by self-resetting. The solid line represents the result of fitting with a typical noise curve. As shown in both Figures 8A,B, the SNR curve follows the illuminated light power, indicating similar behavior on both low and high luminance. The SNR increases by 20 dB in low illuminance and 10 dB in high illuminance. This is consistent with the fact that light intensity independence noise, such as external resetting and pixel readout, is dominant at low illuminance. Conversely, photon shot noise is dominant at high illuminance. The SNR was improved by applying the noise filter described in the next section, and a high effective SNR of 70 dB or more was achieved in both pixels.

Due to the different photodiode structures, the previous version had a smaller capacity. Therefore, in the low-light range, the SNR is higher than that of the new pixel because it has higher sensitivity due to the smaller capacitance of the photodiode. However, this area was not important for our purposes. Alternatively, the effective SNR in the high-illuminance range is approximately the same. The low photodiode capacity makes self-resetting more frequent and causes the device to become unstable. In particular, noise increases owing to dead time by self-resetting and the residual response nonlinearity error. These issues can be mitigated by a lower number of resets. The number of self-resets at a light intensity of 1.35 mW/cm^2^ was 52 for the N-well/P-sub structure and 20 for the P-diff/N-well/P-sub structure. The ratio of PD capacitances in the previous and present sensors is approximately 1:2.6 for the self-resetting period. Consequently, the total effective SNR is approximately the same level, but the latest design provides a more stable device.

### Performance Improvement by the Relay Board

**FIGURE 9:**
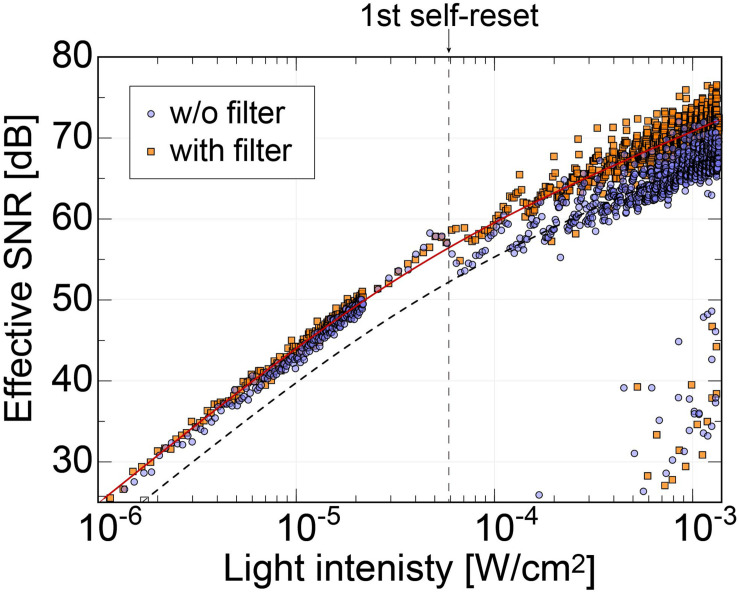
Comparison of the effective SNRs as functions of the incident light intensity between the image sensor using relay boards with and without the noise filter on the Vrst line. The result shows that the SNR dropping behavior at the first self-resetting has been reduced with our latest relay board.

In our previous work, the noise increased significantly at the first self-reset, and since then, it has been a factor that lowers the overall effective SNR (Sasagawa et al., 2016b; Yamaguchi et al., 2016). It was speculated that this was because the reset potential temporarily dropped, and the V_*RST*_ potential did not recover to a constant value until the end of the self-reset. Conversely, the new relay board used in this study is equipped with a noise filter (NFM18PS, Murata) on the V_*RST*_ line. Figure 9 shows the effective SNR difference with and without a filter. The data of the device with the filter are the same as those in Figure 8B. The dashed line represents the curve fitted to the data without the filter. Here, only the offset was changed from the fitted curve for the device with the filter.

Without the filter, there was a significant SNR reduction in the initial self-reset, as in previous devices. However, no significant increase in noise was observed with the filter. As a result, we succeeded in obtaining an SNR improvement of approximately 4 dB. Here, we chose a passive filter to reduce the noise. From the simulation result, the self-reset operation is expected to be completed in 0.1 ns or less. That is, a response bandwidth of approximately 10 GHz or higher is required. It is difficult to achieve such a high-speed response with low-power and active devices. Stable operation is realized by mounting an external filter with a capacity sufficiently larger than the pixel capacity and charging the pixel PD capacity in a short time.

## Imaging Experiment

### Experimental Setup

**FIGURE 10:**
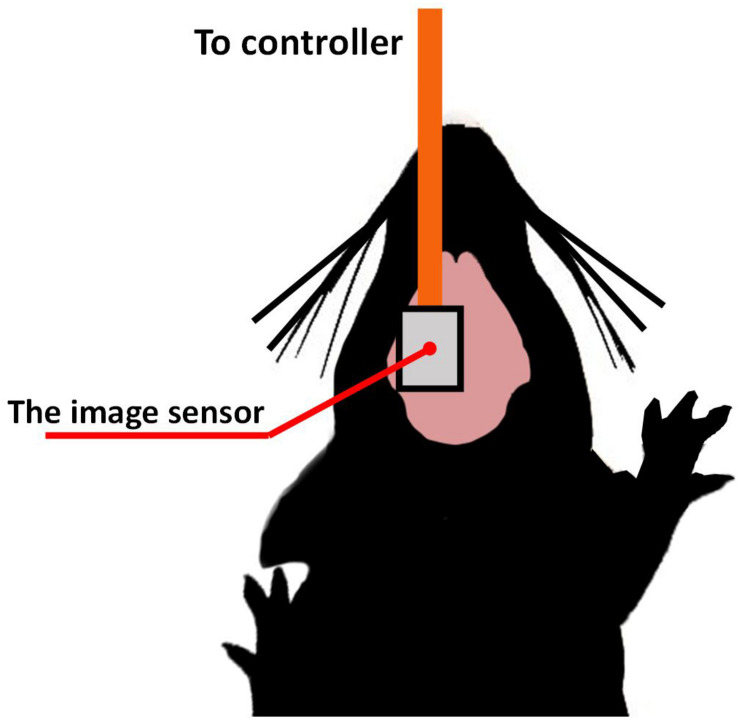
The imaging device is ready to be placed at the left side of the brain on the somatosensory cortex area, while the stimulation was performed on the opposite side.

For the imaging demonstration, intrinsic signal observation was performed on the mouse brain surface. We used wild-type mice (C57BL/6JJmsSlc) from Japan SLC, Inc. The target area was the barrel cortex, a sub-area of the somatosensory cortex. Figure 10 shows a diagram of the experimental setup. Urethane (10%, Wako Pure Chemical Industries, Inc., Japan) was first administered intraperitoneally at a weight-dependent dose (1 g/kg). The use of urethane as systemic anesthesia allows for extended periods of imaging. It is suitable for sensory stimulation studies since neural responses remain relatively unattenuated and are less variable in time across repeats compared to other anesthetics (Pisauro et al., 2013). The head of the mouse was fixed with the stereotaxic instrument. The sensor was placed on the target area brain surface. We chose bluish-green with a center wavelength of 527 nm (SMLP13EC8TT86, ROHM). All animal experimental procedures were controlled by the Nara Institute of Science and Technology’s Animal Care and Experimentation Guidelines.

### Imaging Results

**FIGURE 11:**
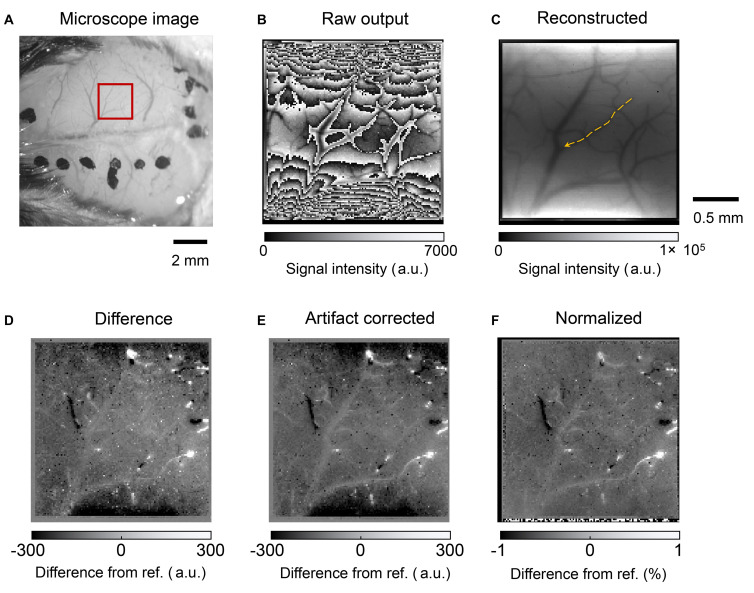
**(A)** Photograph of the mouse brain surface. The red square shows the position where the image sensor was placed. **(B)** Raw output image from the self-reset sensor. **(C)** Reconstructed image. The orange arrow indicates the blood vessel analyzed in Figure 12. **(D)** Difference image from the reference. White and black spots are observed because high output value change occurs when the number of self-resets changes. **(E)** Image after noise and artifact reduction. **(F)** Image normalized by the reconstructed image.

Figure 11 shows the mouse brain images. A photograph obtained using a microscope is shown in Figure 11A. The red square represents the area where the image sensor was placed. Figure 11B shows the raw output image. The frame rate was approximately 15 fps. The LEDs are located on the top and bottom sides of the image. When the light intensity reaches a threshold, the imager resets itself. Thus, the folded intensity fringe pattern appears in the image. This is not a problem for our purpose, that is, to observe the intensity change from a reference image. An important characteristic is the effective SNR for detecting small intrinsic signal intensity changes. In addition, the normal image can be reconstructed by comparing it with a reference image or an estimating algorithm. The number of self-resetting events can be counted when the illumination intensity is gradually increased. The self-resetting number in the central part of Figure 11B is three. The reconstructed image is shown in Figure 11C.

Our target was to clearly obtain different images from the reference image. In this study, we used MATLAB and an image processing toolbox (MathWorks, Inc.). The processing procedure was as follows:

1) Nonlinearity compensation: The present pixel shows nonlinearity that cannot be ignored. The correction curve was prepared from the measured output results vs. incident light intensity by polynomial fitting. Nonlinearity was compensated using the result as a look-up table.
2) Intrinsic signal imaging: The reference frame was subtracted from each frame; the resulting small intrinsic signal difference in each frame can easily be emphasized and visualized. The reference image used was an average of 1000 frames of sequentially acquired data. The results are shown in Figure 11D.
3) Correction of self-resetting artifacts:As mentioned above, self-resetting brings the self-resetting artifact as a complex boundary, as shown in Figure 11B. The number of self-resetting results in a high output change. However, our target signal was a small change. Thus, it can be easily separated by setting an appropriate threshold. Then, it is compensated by adding or subtracting the output swing value to make it shift off from that level. Here, the swing amplitude is slightly different for each pixel. This time, it was set to 99% of the maximum and minimum values in a series of images.
4) Reduction of pulsation and high-frequency noise: The mouse heartbeat was superimposed on the acquired image as a periodic brightness change. For each pixel, a 9–12 Hz component including a mouse beat band and a 15 Hz or higher component, including a high-frequency component, were removed from the frequency spectrum obtained by FFT, and then a waveform was obtained by inverse FFT. The salt and pepper noise that occurs in places at the self-reset boundary is reduced by image processing. An example of the final results is shown in Figure 11E.
5) Pixel normalization: The observation area illumination is not uniform. To compare it, every frame was divided by the reconstructed reference image shown in Figure 11F.

The above results show that the self-reset boundary can be removed by image processing, although a slight noise component remains. Especially in the boundary part, there is almost no significant noise increase because of the number of self-resets. Furthermore, when the pixel output is used as is, a change in the signal strength difference appears on both sides of the self-reset boundary, but it can be reduced to an invisible level by correcting the nonlinearity. This is in agreement with the SNR curve results shown in Figure 8. Significant boundary artifacts owing to self-resets occur when the pixel value that reads the self-reset period overlaps. It is difficult to remove this, and the pixel information with an abnormally large value was removed to manage it in this study.

**FIGURE 12:**
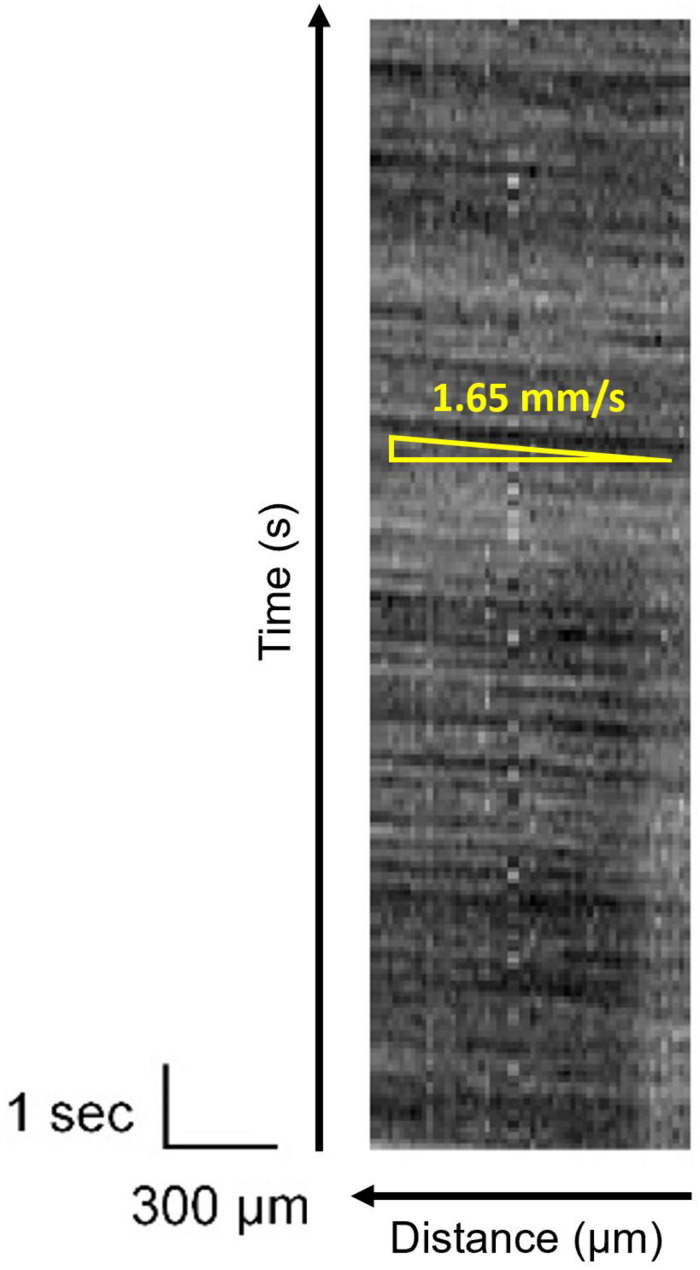
Line scan image of the blood vessel indicated in Figure 11C. The movement of red blood cells is shown as dark stripes.

In the intrinsic imaging, the concentration of blood in a target area is observed, and no information is obtained from the blood vessels. However, the red blood cell flow can be observed in the vessels. It allows estimating blood flow velocity, which is correlated to brain activity (Kleinfeld et al., 1998; Bouchard et al., 2009; Haruta et al., 2014). Figure 12 shows the temporal change in the brightness of the blood vessel, as shown in Figure 11C. It is a line scan along with the blood vessel and plotted as a function of time. This dark stripe corresponds to the higher number of red blood cells. It shows the distance that the red blood cells can travel in time, which reflects the frame rate. The blood velocity can be calculated by dividing Δ*x(distance)* by Δ*t(time)*. Because of the improvement of the effective SNR, the stripe pattern has been observed clearly. Thus, an oblique pattern owing to flow velocity in the blood vessels was observed. The observed blood flow velocity was 1.4–2.8 mm/s. When there is a pulsation, periodic vertical stripes are formed, but this can be clarified by reducing the pulse noise by image processing.

## Discussion

### Comparison With Other Sensors in the Previous Works

The proposed pixel offers a small size with a high fill factor. Compared to its previous version (Yamaguchi et al., 2016), a base prototype, the SNR is up to 64 dB. With the modified photodiode structure, we succeeded in increasing the SNR to over 70 dB even when the other parameters remained constant, such as pixel size and fill factor. Moreover, this larger FWC strategy also offers a more stable operation. Because a larger FWC reduces the number of self-resets, it can avoid the unstable stage. In particular, compared to other image sensors with a similar self-resetting function (Leñero-Bardallo et al., 2017). Because our pixel has a small circuit part, it is possible to realize a fill factor of the same level or higher, even though it is a relatively small pixel as compared with other self-reset pixels. The proposed image sensor was designed to monitor the brain activity. Thus, the high dynamic range is not as important in that the intensity change is of interest. What matters is the high SNR to detect a relatively small intensity change in brain activities. The effectiveness of sensing the illumination is important in that it can avoid using too high a light intensity to achieve the duty, which can cause the temperature to increase and damage the brain. In addition, methods for estimating the original image from the self-reset sensor image group without a counter have been proposed, and it is possible that these methods will expand the range of applications (Bhandari et al., 2017).

For SNR, Murata and Fujihara et al. presented a pixel with a high SNR of 70 dB (Murata et al., 2020; Fujihara et al., 2021). The pixel size was 16 × 16 μm, and ultra-large capacitances were achieved with in-pixel capacitors. The sensor has a high dynamic range and SNR. However, this requires a special capacitor fabrication technology. Our proposed chip can achieve a similar SNR with a small implantable chip fabricated using a 0.35-μm standard CMOS process. The fill factor can be further increased using a finer process.

### Limitations of Pixel Performance

Regarding the frame rate, from the simulation results shown in Figure 2, it is estimated that the self-reset artifact can be neglected even if the current pixels are approximately five times faster (∼150 fps). However, for applications that require a high SNR and frame rate, such as voltage-sensitive dye imaging, the self-resetting duration should be shorter. To manage this, a frame rate of approximately 1 kHz or more is required, which is insufficient under the current conditions. However, the simulation predicts that if V_*b*_ is changed to shorten the self-reset time, the V_*PD*_ will not be firmly reset until V_*RST*_. To achieve high-speed operation, it is necessary to make changes such as increasing the reset transistor size.

From this study, it was found that the pixel output nonlinearity with respect to the light intensity can be reduced by correction using the pixel output characteristics acquired in advance. However, when the brightness changes significantly, there are cases in which the error increases. This error can be significantly reduced by improving the nonlinearity. For that purpose, charge transfer to a highly linear capacitance, similar to a general 4-transistor type APS pixel, can be considered.

### *In vivo* Imaging Results

By using FOP in the manufactured device, an almost sufficient spatial resolution was obtained with a pixel size of 15 μm square. In addition, almost no additional noise owing to being mounted on a living body was observed. For the pulsating noise, the frequency component was removed using an FFT. Clear improvements were observed in the captured images, as shown in Figures 11D–F. The range of brightness is ±1%, and the results show that slight changes in brightness can be observed. However, this technique can only be used for post-processing. To observe in real time during the experiment, it is necessary to introduce a digital filtering technique.

In Figure 12, owing to the high SNR, the change in brightness because of the red blood cell concentration in the blood vessels is clearly visible. In this experiment, the frame rate was set to 15 fps. The resolution of the flow velocity can be improved by increasing the light source brightness and by improving the frame rate. However, it is necessary to consider the heat effects on the observation target.

## Conclusion

We succeeded in designing and fabricating an image sensor with a self-resetting system, which has an SNR exceeding 70 dB. We increased the pixel capacity to reduce the self-reset frequency and improve operational stability. Moreover, additional noise by self-resetting was decreased by approximately 4 dB after stabilizing the reset voltage during self-reset. Furthermore, image processing reduced artifacts near the boundary of the number of self-resets. The prototype imaging device was applied to the intrinsic signal imaging of a brain surface.

With this device, a high SNR was achieved with a small device that can be mounted on the mouse head. By applying it not only to intrinsic signal imaging but also to voltage-sensitive dye imaging (Ferezou et al., 2006; Tsytsarev et al., 2014), it is expected that it can be applied to brain function observation associated with various behaviors.

## Data Availability Statement

The raw data supporting the conclusions of this article will be made available by the authors, without undue reservation.

## Ethics Statement

The animal study was reviewed and approved by the Nara Institute of Science and Technology (NAIST) Animal Committees.

## Author Contributions

TP contributed to chip design, device fabrication, data analysis, and writing the manuscript. KS contributed to the conception, design, data processing and interpretation, and writing the manuscript. MG contributed to animal experiments and data analysis. YO contributed to the animal experiments. MH, HTk, HTs, and JO contributed to interpretation of the obtained data significance. All authors contributed to the manuscript and approved the submitted version.

## Conflict of Interest

The authors declare that the research was conducted in the absence of any commercial or financial relationships that could be construed as a potential conflict of interest. The handling Editor declared a past co-authorship with the authors.
